# Recognition and Localization Methods for Vision-Based Fruit Picking Robots: A Review

**DOI:** 10.3389/fpls.2020.00510

**Published:** 2020-05-19

**Authors:** Yunchao Tang, Mingyou Chen, Chenglin Wang, Lufeng Luo, Jinhui Li, Guoping Lian, Xiangjun Zou

**Affiliations:** ^1^College of Urban and Rural Construction, Zhongkai University of Agriculture and Engineering, Guangzhou, China; ^2^Key Laboratory of Key Technology on Agricultural Machine and Equipment, College of Engineering, South China Agricultural University, Guangzhou, China; ^3^College of Mechanical and Electrical Engineering, Chongqing University of Arts and Sciences, Chongqing, China; ^4^College of Mechanical and Electrical Engineering, Foshan University, Foshan, China; ^5^Department of Chemical and Process Engineering, University of Surrey, Guildford, United Kingdom

**Keywords:** vision, agricultural harvesting robotic, 3D reconstruction, fault tolerance, recognition, classification

## Abstract

The utilization of machine vision and its associated algorithms improves the efficiency, functionality, intelligence, and remote interactivity of harvesting robots in complex agricultural environments. Machine vision and its associated emerging technology promise huge potential in advanced agricultural applications. However, machine vision and its precise positioning still have many technical difficulties, making it difficult for most harvesting robots to achieve true commercial applications. This article reports the application and research progress of harvesting robots and vision technology in fruit picking. The potential applications of vision and quantitative methods of localization, target recognition, 3D reconstruction, and fault tolerance of complex agricultural environment are focused, and fault-tolerant technology designed for utilization with machine vision and robotic systems are also explored. The two main methods used in fruit recognition and localization are reviewed, including digital image processing technology and deep learning-based algorithms. The future challenges brought about by recognition and localization success rates are identified: target recognition in the presence of illumination changes and occlusion environments; target tracking in dynamic interference-laden environments, 3D target reconstruction, and fault tolerance of the vision system for agricultural robots. In the end, several open research problems specific to recognition and localization applications for fruit harvesting robots are mentioned, and the latest development and future development trends of machine vision are described.

## Introduction

The field of robotics is broad and covers several diverse technological areas (Yang et al., 2018). Machine vision applications enable robots to actively and accurately identify and locate targets. Robotic and automated systems are currently being developed to accomplish work done by operators in the industry, medicine, and military fields (Li et al., 2017; Daudelin et al., 2018). Recent technology advancement in visual identification and 3D reconstruction, positioning and fault tolerance increased the applications of robotics in agriculture including crop harvesting. Like other robotic systems in the field, agricultural robots use artificial intelligence to perform various labor-intensive agricultural tasks such as planting, spraying, trimming and harvesting (Edan et al., 2009; Zhao et al., 2016; Zhang et al., 2019). In many developing countries that are highly dependent on agriculture for food, employment, income, and social stability, agriculture harvesting robots have become an urgent need. With increasing urbanization and shortage of labor, the application of agriculture harvesting robots has the potential to increase productivity, reduce waste, and improve agriculture sustainability.

Crops vary significantly in shape, size, color texture and other physical, chemical and nutritional properties. Of many agriculture crops, fruits are economically essential and have the highest nutritional and health benefits. Fruits have biological characteristics depending on their growth environment, spatial position, geometric shape, size, color, and hardness. Fruit harvesting is a mechanical and repetitive job that is time-consuming labor-intensive. These reasons have prompted research into fruit picking robots (Watts et al., 1983; Ceres et al., 1998; Van Henten, 2006; Van Henten et al., 2009; Zou et al., 2012, 2016; Hiroaki et al., 2017; Xiong et al., 2018a, b, 2019). Several machine vision-based agricultural harvesting robots have been developed (Sarig, 1993; Kendo et al., 1996; Bulanon et al., 2002, 2004; Hayashi et al., 2002; Van Henten et al., 2003; Grift et al., 2008; Scarfe et al., 2009; Yin H. et al., 2009; Bechar, 2010; Li et al., 2010, 2011; Wibowo et al., 2016; Yu et al., 2018), based on advancements in visual recognition and position detection, segmented fruits and their associated algorithms, and reconstructed 3D fruits by stereo matching to calculate the spatial coordinates of fruit targets. The main challenges for robotic subsystems include hands-free navigation and fruit localization (Jiménez et al., 2000b; Li et al., 2009; Kapach et al., 2012; Wang, 2018; Blok et al., 2019).

Most fruit fields have rough terrain with large obstacles, causing heavy vibration in the vision system of mobile harvesting robots as they traverse the terrain. This complication requires the use of dynamic target tracking and automatic image de-blurring algorithms. The topic attracted the attention of interdisciplinary researchers. Tang et al. (2018) use binocular vision to track vibrations caused by terrain deformation and to detect the 3D deformation surface. For example, when wild fruit trees are blown by the wind and interfere with the mechanical arm, the robot’s vision system experiences vibrations, which cause imprecise target-tracking and imaging. Besides, binocular vision is applied to detect the vibrations caused by target movement. A spatial coordinate error model, together with a comprehensive compensation model is established. The robot’s fault-tolerant technology is tested via virtual and physical robots (Zou et al., 2012).

A harvesting robot is designed to pick fruits automatically under certain environmental conditions. Research on harvesting-robot-based machine vision is still in its infancy. With the development of artificial intelligence technology, 3D spatial information about the target can be obtained and processed. Stereo vision technology is a major bottleneck in harvesting robot applications (Zou et al., 2012; Gongal et al., 2015), especially in crop identification, localization algorithms, error handling and small object dynamic tracking.

Here we report on fruit recognition and localization algorithms in detail by examining the following three aspects. First, the visual sensing technology; this is where stereoscopic fruit recognition and localization algorithms are expounded. Then, three techniques are used to explain how stereo vision recognizes and locates fruits under different environmental conditions. Finally, an algorithm based on 3D reconstruction is reviewed. The algorithm provides the spatial coordinates of fruit so that the robot can harvest it. Visual fault tolerance is an essential step in the successful harvesting of fruit in locations with rough terrains, which is rarely seen in most review articles. The performance indicators of the references are listed in Table 1. The “/” symbol signifies that the reference has not provided an indicator for readers or the indicator is difficult to be concluded from the literature.

**TABLE 1:** Essential performance indicators of the introduces studies in this paper.

**References**	**Recognition accuracy (%)**	**Recognition time (s)**	**Harvesting success rate (%)**	**Harvesting time for per fruit(s)**
Zou et al. (2016)	85–94	0.8	84–88	11.3–15.5
Liu et al. (2019)	93.5	/	/	/
Hemming et al. (2014)	90	/	/	/
Qingchun et al. (2012)	/	/	86	10
Zhao et al. (2011)	/	/	77	15
Bulanon et al. (2010)	88–93	/	/	/
Zhao et al. (2016)	93	/	/	/
Wang Z. et al. (2017)	81.1–100	/	/	/
Tanigaki et al. (2008)	83.3	/	66.7	14
Barth et al. (2019)	73	/	52	/
Kondo et al. (2009)	/	/	73	/
Xiang et al. (2014)	87.9	0.5	/	/
Si et al. (2015)	89.5	/	/	/
Makky et al. (2013)	65–70	/	/	/
Wang et al. (2016)	97.5–98.8	/	/	/
Luo et al. (2016)	87	0.7	/	/
Jimenez et al. (1999)	/	/	/	/
Bac et al. (2014b)	94	/	/	/
Peng et al. (2014)	95	/	/	/
Wei et al. (2014)	95	/	/	/
Wang C. et al. (2017)	87.3–93.6	/	/	/
Reis et al. (2012)	91–97	1.5	/	/
Hayashi et al. (2005)	85	43.2	29.1	/
Arefi et al. (2011)	96	/	/	/
Hannan et al. (2007)	90	/	/	/
Kong et al. (2010)	90	0.19–0.27	/	/
Tao et al. (2014)	73.93	1.1	/	/
Dey et al. (2012)	96–98	/	/	/
Hannan et al. (2009)	90	/	/	/
Yamamoto et al. (2014)	88	/	/	/
Kurtulmus et al. (2011)	75.3	/	/	/
Xu et al. (2019)	/	0.59	/	/
Li et al. (2012)	90.15	/	/	/
Kim et al. (2014)	90	/	/	/
Zhang et al. (2008)	87.6	/	/	/
Yang et al. (2016)	86.5	/	/	/
Fu et al. (2018)	92.3	/	/	/
Sa et al. (2016)	80.7	0.3	/	/
Horea and Mihai (2018)	96.3	/	/	/
Luo et al. (2018)	81.66	0.53–0.69	/	/
Lin et al. (2019)	88	0.59	/	/
Gongal et al. (2016)	82	/	/	/
Ehud et al. (2016)	55	/	/	/
Williams H. A. M. et al. (2019)	76.1	3	51.0	5.5
Lee et al. (2019)	82.16	/	/	51.1
Tao et al. (2017)	80.34–92.3	/	/	/
Majeed et al. (2018)	88–93	/	/	/
Sa et al. (2017)	71	/	/	/
Silwal et al. (2016)	100	1.6	/	6.1
Liu et al. (2018)	95.35	/	/	/
Williams H. A. M. et al. (2019)	/	/	86	2.78
Vitzrabin and Edan (2016)	/	/	79	/
Onishi et al. (2019)	90	2	/	16
				

## Visual Harvesting Robot

The working environment of the visual components of a fruit harvesting robot is very complicated. The working objects are the crops, which include apple, litchi, citrus, grape, strawberry, or sweet pepper. These objects vary in size, shape, color, and texture. The background and illumination of the crops vary continuously (Bulanon et al., 2010; Zhao et al., 2011; Qingchun et al., 2012; Hemming et al., 2014; Silwal et al., 2016; Liu et al., 2019; Williams H. et al., 2019; Zhuang et al., 2019). Machine vision-based harvesting robots should have the ability to sense and adapt to different crop types or environmental changes (Zhao et al., 2016; Silwal et al., 2017), collect information, detect targets, and learn autonomously. The robots should also be able to apply intelligent reasoning and engage in decision making. It is an intelligent automated machine for human-computer interaction (Kondo and Ting, 1998; Zou et al., 2012; Zhuang et al., 2019). The robotic system should also have a network transmission function for sending the crop images to a data center or server (Garcia-Sanchez et al., 2011). Agricultural robotic systems have similar structures and are composed of an autonomous mobile platform, a light multi-degree-of-freedom mechanical arm, a force feedback system with a flexible end effector, a multi-sensor machine vision system, a drive control system, an intelligent decision system, and auxiliary software and hardware.

**FIGURE 1:**
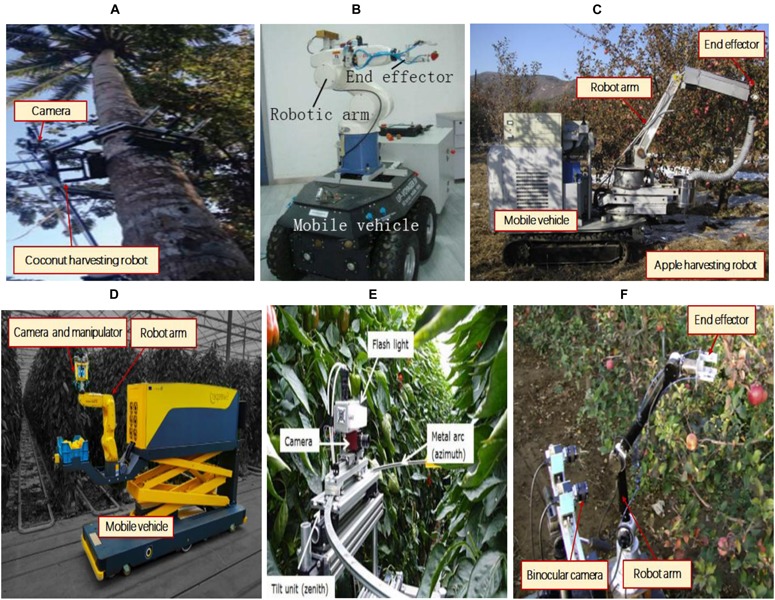
Representative forms of picking robots in the references. **(A)** Autonomous coconut-harvesting-robot (Wibowo et al., 2016); **(B)** Strawberry harvesting robot (Qingchun et al., 2012); **(C)** Apple harvesting robot (Zhao et al., 2011); **(D)** Sweet-pepper harvesting robot (Barth et al., 2019); **(E)** Another sweet-pepper harvesting robot (Hemming et al., 2014); **(F)** Another apple harvesting robot (Si et al., 2015).

The first task of a fruit harvesting robot is to use visual sensing to perceive and learn crop information (Zou et al., 2012; Zhao et al., 2016). Its tasks include camera calibration (Wang et al., 2019), target recognition and positioning, target background recognition, 3D reconstruction, visual positioning-based robot behavior planning, mechanism, and vision. The system is also collaborative and uses a visual servo-control picking mechanism to perform clip-cutting operations. Changes in the illumination of complex agricultural crop environments, vibrations caused by wind or manipulators, and inaccurate positioning caused by a variety of uncertain factors (Jiménez et al., 2000a; Gongal et al., 2015; Wang C. et al., 2017; Xiong et al., 2018b) can result in harvesting failure. This is the leading technical challenge in the development of visual systems for agricultural harvesting robots. Figure 1 lists some representative forms of picking robots.

**FIGURE 2:**
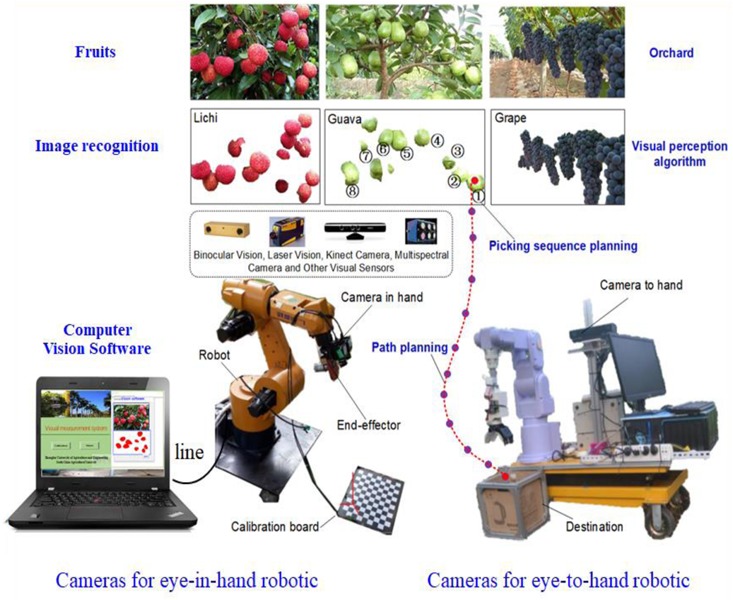
Vision-based fruit picking robot system.

The vision system of fruit harvesting robots (see Figure 2) has several sensing capabilities (e.g., visual sensing, collaborative visual-mechanical control, visual recognition, 3D reconstruction, coordinated visual-mechanical positioning and error tolerance). Visual sensing focuses on the image-based data collection of crops (Jiménez et al., 2000a). Vision-based target recognition uses various perception modalities and an accurate recognition scheme. For example, fruit imaging algorithms divide the background into a series of features (Bulanon et al., 2010). Background features, including branches, leaves, and adjacent fruits, are obstacles for mechanical operation, affecting the fruit-picking behavior of the robot. The area around the target can be labeled the target space and used for 3D reconstruction of the target. Visual-institutional coordination and error tolerance can be used to enhance robotic anti-collision precision picking guidance, picking sequence planning and the decision-making behavior of a robot (Tanigaki et al., 2008; Zou et al., 2012; Barth et al., 2019; Zhang et al., 2019).

**FIGURE 3:**
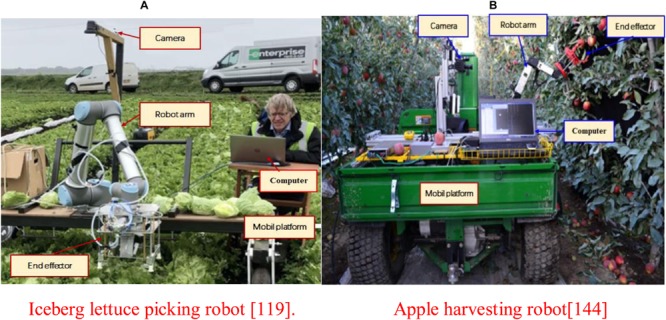
Multi-DOF fruit and vegetable harvesting robot and its vision system for field operation. **(A)** Iceberg lettuce picking robot (Birrell et al., 2019). **(B)** Apple harvesting robot (Silwal et al., 2017).

The robot arm of a common vision harvesting robot has at least 6 degrees of freedom (DOF); this ensures that the robot’s movements are flexible. For example, Birrell et al. (2019) designed an iceberg lettuce picking robot (see Figure 3A). They used a deep detection network to roughly locate the iceberg lettuce, to achieve accurate identification and to harvest the iceberg lettuce via the device. The robot has a harvest success rate of 97% and a harvest time of 31.7 ± 32.6 s. To increase the robot’s flexibility, Kondo and Shunzo (1989) and Kondo et al. (2009) studied a tomato picking robot with 7 degrees of freedom and developed a visual recognition algorithm that could identify individual fruits and bunches. The picking time of a single fruit is about 15 s and the success rate is about 70%. Silwal et al. (2017) designed an advanced seven degree of freedom apple harvesting robot with precise positioning capability (see Figure 3B). The average positioning time of each fruit is 1.5 s, the average picking time is 6 s per fruit and the picking success rate is 84%. This robot has fast speed and can meet the needs of farmers. Adopting seven degrees of freedom improves the robot’s flexibility and obstacle avoidance. However, the orchard environment was highly controlled, e.g., the clusters of fruit were removed, which reduced the complexity of the environment.

In general, the basic structure of the picking robot has been formed. However, the success rate of most harvesting robot prototypes has been around 66% (values ranging between 40 and 86%) with a cycle time of 33 s per fruit (values ranging between 1 and 227 s) (Arad et al., 2020). These measures of performance indicate that robotic harvesting technology performance is still low. One of the main factors restricting the development of harvesting robots is determining an accurate three-dimensional visual perception and the stability of machine operations in complex environments. Therefore, most of the research has focused on improving the stability and reliability of robotic functions so that the harvesting robot can cope with various complex agricultural operating environments.

In order to evaluate the overall performance of harvesting robots, Bac et al. (2014b) reviewed 50 harvesting systems and summarized their average performance: location finding (85%), fruit detachment (75%), harvesting (66%), and rate of fruit damage (5%). Some researchers have considered the use of “cycle time” to evaluate the potential of a research study to be transformed into a commercial product, which includes the entire process from the start of the robotic work to the successful harvesting of fruit. As of 2014, the average release cycle of non-industrial systems was 33 s (Bac et al., 2014b; Williams H. A. M. et al., 2019). However, this measure has not been widely used. In fact, for different agricultural crops, the performance parameters that a harvesting robot can achieve are different (see Table 1). Commercial practitioners estimated that for kiwifruit, at least 80% of the fruit in a canopy needs to be collected at an average rate of four fruits per second (Williams H. A. M. et al., 2019). According to our survey, users of litchi harvesting requires that the picking time of a single bunch of litchi should not exceed 15 s, and the picking rate should reach about 70%; while for tomatoes, the picking time of each fruit should be 5–8 s, and the picking rate should reach 90%. So far, there are no unified and clear indicators to measure the comprehensive commercial performance of harvesting robots, which needs further discussion and research.

## Visual Sensing Technology

The visual sensing technology of harvesting robots is designed to detect crops and fruits. A robotic servo controller collects 3D information about the environment surrounding the fruit, including geometry and 3D coordinates. The visual camera and its control system can serve as the hardware support of the visual sensing technology, which serves as a communication interface between the external environment and the robot. Images obtained by cameras are generally classified into digital images, laser images, and multi-spectral images. This section will review the overall visual sensing technology and its components (see Figure 2).

### Stereo-Vision Systems

Currently, two forms of stereo vision systems are mainly deployed. The first is a binocular vision system based on optical geometry. The 3D position of the target is obtained through traditional optical principles and optimization algorithms. The second is an RGB-D camera based on the time-of-flight (ToF) method, which uses an infrared sensor to obtain the depth information of the target. The ToF method is sensitive to external interference and may not work in the scene with strong light. On the other hand, the depth measurement accuracy of this method is limited by the working distance of the infrared sensor. By contrast, the optical geometry-based method is a passive measurement method, which does not rely on artificial light sources and can be used in indoor and outdoor environments. Therefore, to ensure stability in agricultural picking tasks, a binocular vision system based on optical geometry is needed. Since the principle of the RGB-D camera is simple and the system is compact, it can be used for many local tasks, such as three-dimensional reconstruction of targets at specific locations. Therefore, some brief introduction of the application of RGB-D cameras will also be briefly introduced in section 3D Reconstruction Method for Vision-Based Target, but not in this section.

The optical geometry based stereo-vision system consists of two or more cameras separated by a fixed distance (Zou et al., 2012; Zhao et al., 2016). Before the detection process, the cameras are calibrated. First, two or more images of the same target are obtained via stereo vision. The images are processed and classified to identify the target object. The 3D target is reconstructed by relating the spatial coordinates of the target to those of the robot. This relationship provides the physical parameters needed to achieve target identification and localization.

The binocular stereo vision detection technology is based on monocular vision. Early monocular vision systems used a single camera to detect one two-dimensional image of the target. Separate image analysis is performed to identify its features. With the development of computers, scholars in the 1960s began to explore theoretical research on 3D images and stereoscopic machine vision (Roberts, 1965). The application of target detection in crops has also undergone a transformation from two-dimensional to 3D vision. Schertz and Brown used light information testing for fruit harvesting robots as early as the 1960s (Brown and Schertz, 1967). Since the 1980s, the monocular vision was a standard component of agricultural robots. The monocular vision was used to detect the two-dimensional geometric features of crops, to identify red fruits and green leaves by detecting geometric shapes and color features. This feature detection process has a detection accuracy of about 75% (Harrell et al., 1985; Slaughter et al., 1986; d’Grand et al., 1987; Slaughter and Harrell, 1987; Kondo and Shunzo, 1989; Xue et al., 2012). As the sensing modalities and algorithms became sophisticated, researchers began to examine the role of light to obtain information about crops (Kondo et al., 2009). These tasks often required multiple monocular cameras (Edan et al., 2000).

The binocular stereo vision was first used in agricultural harvesting robots to identify tomatoes, sweet peppers, and apples (Buemi et al., 1996; Kitamura and Oka, 2005; Xiang et al., 2014). Plebe and Grasso (2001) installed stereo cameras in each arm of an orange harvesting robot. Stereo matching of the oranges’ center-of-mass was performed to locate oranges in a 3D coordinate system. Si et al. (2015) used a stereo camera to detect and locate mature apples under a canopy. The authors reported that over 89.5% of apples were successfully recognized and the errors were less than 20 mm when the measuring distance was between 400 and 1,500 mm.

Makky and Soni (2013) proposed a stereoscopic 3D vision sensing system for a palm oil-collecting robot project. The team obtained two stereo images using a mobile digital camera and used image processing algorithms for target recognition, thereby detecting palm fruit-based on image color analysis and fruit maturity-based features. The method can calculate the distance, size, and tangential position of the palm fruit. For red fruit dense images and yellow-green apple images, the fruit recognition rate was between 65 and 70%, with a ranging error of ∼±5% (Takahashi et al., 2000). For stereoscopic detection of a single fruit, the system needed to determine the center-of-mass coordinate of the fruit first so that the robot’s fingers could grip the fruit and twist the fruiting branch. This operation of griping and twisting fruit is relatively straightforward using visual inspection. No detection of the mother branch is necessary for pinching the fruit; nevertheless, it can easily pick the fruit by using a pinching action.

**FIGURE 4:**
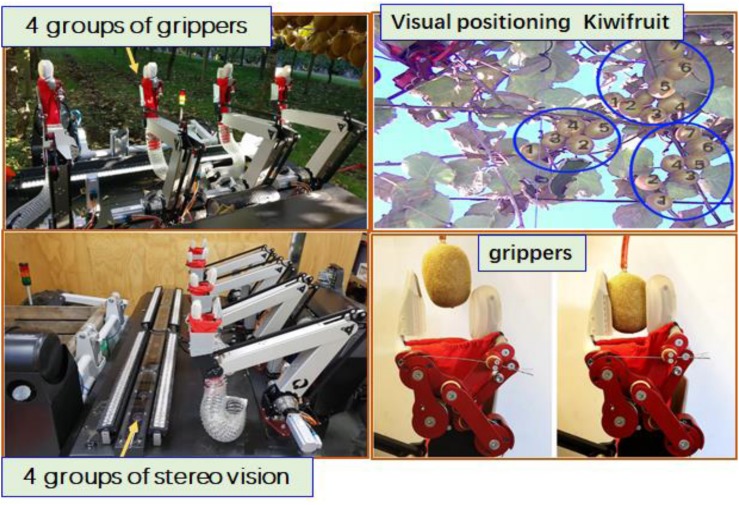
Kiwifruit harvesting robot based on stereo vision (Williams H. A. M. et al., 2019).

To improve the picking speed of the harvesting robot, Williams H. A. M. et al. (2019) studied the kiwifruit harvesting robot with four arms (see Figure 4). Each robotic arm has a corresponding set of binocular vision to detect fruits and locate their positions in 3D space. It takes 3 s to process the complete image, and the visual recognition success rate is 76.3–89.6%. The harvesting robot mechanism is flexible in design, and the four arms and four pairs of binocular vision can work cooperatively with high efficiency. However, about a quarter of the fruit still fell to the ground during the picking process. This was mainly due to interference from obstacles, which caused positioning errors or small friction between the robotic fingers.

**FIGURE 5:**
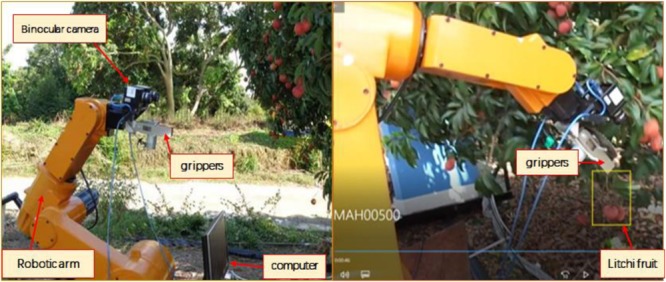
Litchi picking robot based on binocular stereo vision.

Zou et al. (2016) proposed an end effector with certain versatility to clamp and cut the fruit with fault-tolerant design. The eccentric cutter is installed above the clamp so that the damage to the fruit and its body is minimal(see Figure 5). The picking method makes stereoscopic vision detection more difficult. In addition to detecting and identifying the fruit, it also identifies and estimates the spatial position and coordinate points (also called picking points) needed to determine the clamping and cutting points of the mother branch. Two color-cameras were mounted on a six-degree-of-freedom robot that was used to locate the litchi in an unstructured environment. The litchi fruit was extracted by stereo matching two litchi images in the same scene. The recognition method is robust to changes in illumination, so 3D information can be used to recognize litchi fruits accurately. The average recognition rates of unobstructed litchi and partially occluded litchis were 98.8 and 97.5%, respectively (Wang et al., 2016). A binocular vision-based recognition of grapes was also investigated. The feature matching and localization capabilities of the robot to detect grapes and their picking points were shown. Due to the complexity of the terrain, the visual and stereo matching modalities are disturbed by noise, which increased the positioning error (Luo et al., 2016).

The challenges can be identified as follows.

1. In dynamic environments, the shape of the fruit image is inconsistent with the initial shape acquired by the camera, which results in a large error in 3D positioning.
2. In 3D vision technology, the calculation amount of stereo matching is great, which makes it somehow inefficient in real tasks. This is also one of the consensuses in the 3D vision field.
3. With an unstructured environment, illumination and occlusion can affect the accuracy of 3D fruit detection.

### Laser Active Vision Technology

Jimenez et al. (1999) proposed a preliminary method that used a laser-based machine vision system for automatic identification of fruits. The method uses an infrared laser ranging sensor to detect the 3D position, radius, and surface reflectivity of each spherical fruit. Combined with the proposed image analysis algorithm, the method can be applied to the citrus harvesting with ∼80–90% detect rate. Slaughter et al. (1986) proposed an active triangulation-based ranging system consisting of several independent laser systems. Each laser system produces a light scattering sheet that is projected onto the object. Besides, two cameras were used to solve the occlusion problem. This method integrates multiple distance measurements (obtained from these cameras) into a single image to produce very accurate depth measurements. Kondo et al. (2009) applied laser range finder to tomato, cucumber, and grape harvesting robots. A similar scheme for detecting mature cherries using 3D sensors was reported. The results showed that 10 of the 12 fruits were identified.

There are still problems with the use of structured light, such as complex equipment installations, unpredictable occlusions, and indirect measurements. For example, in some cases, the laser may be blocked by obstacles and cannot be projected onto the target. On the other hand, the laser may be out of focus due to long distance and result in fewer features. Furthermore, installing a laser will increase the structural complexity of the entire vision system, which is undesirable in field conditions.

### Multi-Spectral Imaging Technology

The multi-spectral imaging technology can image in different color spectra to see details that are invisible to the naked eye and ordinary cameras. Multi-spectral techniques divide the incident full-band or wide-band optical signal into a number of narrow-band beams, and then image the beam over the sensor. Its agricultural applications include pest and disease monitoring of fruits and crops, and growth assessment of agricultural targets. Multi-spectral technology has excellent research in the field of fruit picking. Liu and Liu (2007) designed a system based on multi-spectral vision technology and triangulation technology to obtain the spatial position and maturity information of apple fruit through a specific optical path, which was an attempt to have great application value; Lu et al. (2011) used multi-spectral imaging techniques to identify branches under different lighting conditions, which ensured the efficiency of path planning and safety of the operation of citrus picking robots in complex natural scenes. Bac et al. (2014a) used different bands to image segmentation, feature extraction and classification of sweet pepper, which provided meaningful reference data for constructing obstacle maps of fruit picking robots. Hung et al. (2013) performed image segmentation using features extracted from multi-spectral data to provide a yield estimation method for fruit differences, and the algorithm reached high accuracy and robustness. Navia et al. (2016) used an automatic four-axis drone with a multi-spectral camera to capture multi-spectral images of terrain and assemble topographic mosaics to measure and evaluate green vegetation. This article provides an integrated drone solution to capture multi-spectral images with geotagging, which has important reference value in terrain and scene perception. Fischer et al. (2019) combined multi-spectral and drone technology and used feature extraction algorithms to detect cornfield areas damaged by wild boar. This yields a new monitoring technology that effectively acquires terrain information and assesses the damage of crops based on its description. Khaliq et al. (2019) obtained multispectral images of vineyards using a ten-meter-resolution satellite and a low-altitude drone platform. The system calculated three different NDVI indices and compared drone data with satellite images to analyze unbundled spectral contribution of different elements in a vineyard. The vigor map, calculated from drone images, has a higher correlation with on-site assessment compared with satellite images, revealing that the multi-spectral and drone platform can accurately complete the tasks of terrain and crop perception.

The multi-spectral technology can provide satisfactory positional and biological information for fruit harvesting tasks and is a strong auxiliary means of vision-based picking frameworks. Since multi-spectral cameras still follow the basic rules of optical imaging, they have the common disadvantages of RGB cameras, e.g., the sampling quality may be affected by changing and uneven light.

## Object Recognition Method for Harvesting Robot

### Single Feature Vision Method and Improvement

The color of crops has significant and stable visual characteristics that are less dependent on the size of the image itself. In machine vision technology, an essential part of image processing is the image segmentation algorithm.

Image segmentation predicts information based on each pixel in an image. Image segmentation has two technical aspects; one is to predict only the segmentation at the class level and to mark the position for each pixel. The second is to distinguish individual objects from a set of objects (Fu et al., 2019). The color characteristics of the fruit are extracted by combining multiple color spaces such as HIS, L^∗^a^∗^b^∗^ and LCD (Yin J. et al., 2009; Cubero et al., 2014).

The algorithm still needs to be improved when the vision is detected in the orchard. Wei et al. (2014) proposed an improved OSTU algorithm based on a fruit recognition scheme that detects and uses fruit color, e.g., red tomato and yellow persimmon would be two distinct targets. The accuracy rate of this algorithm was about 95%. However, robustness is reduced when segmenting with color features, i.e., this method is sensitive to changes in the field environment, especially in the wild.

For the identification of wild crop targets, Peng et al. (2014) proposed a Double Otsu Algorithm to segment litchi fruit orchards and achieved a correct recognition rate of ∼95%. To alleviate different illuminations, Wang Z. et al. (2017) proposed a robust image segmentation algorithm that detected illumination changes. Red litchi, purple grapes, and yellow citrus were used as examples to conduct field experiments; the detection rates were 93, 95, and 88%, respectively (Zhuang et al., 2019). When the color of the fruit is similar to the leaf color, the color characteristics are not significant, and a color-based segmentation method alone cannot be used to identify the fruit (Reis et al., 2012). Thus, it is necessary to combine multiple algorithms to segment the desired target.

Image shape features are mainly derived from the geometric features of the target. There are many typical feature extraction algorithms. These pre-existing algorithms often need to be improved/amended when segmenting images. The algorithm is usually unaffected by changes in illumination and is suitable for field target recognition. Plá et al. (1993) proposed an early single feature analysis method for citrus recognition. The method uses a circular feature to segment the citrus fruit, but the recognition rate is only 50%.

We analyzed individual images according to the geometric features of the target for the round or long-shaped fruits. The Canny operator and Hough transform algorithms are used to detect the contour of the target. This operator-transform pair was used to identify tomatoes, apples, and citrus (Hayashi et al., 2005; Kondo et al., 2009; Zhao et al., 2011; Mehta et al., 2014).

Arefi et al. (2011) combined color space and fruit geometry. The accuracy of tomato recognition in artificial greenhouses was ∼96%. Hannan et al. (2007) proposed a citrus detection method that combined a shape analysis technique and an orange detection algorithm. Experimental results show that more than 90% of the fruits in the 110 images were detected. Kong et al. (2010) used the color feature vector to train the LS-SVM model for apple recognition. The results showed that the recognition rate of apples can reach over 90%. Liu et al. (2019) proposed that under Y’cbcr color space, a visual system was designed by using mathematical models such as elliptic boundary model and regional opening mathematical morphology model to judge whether pomelo was mature or not, and the total accuracy of the algorithm reached 93.5%. Arad et al. (2019) proposed the controlled illumination acquisition protocol for flash-no-flash (FNF). At the same time, a controlled illumination acquisition protocol was obtained for one Flash and one non-flash image. The color-based algorithm was shown to obtain a maximum of 95% precision at a 95% recall level for FNF images, compared to 99% precision at a 69% recall for Flash-only images; this suggests the proposed FNF is effective for color-based detection algorithms.

There are still challenges with the use of structured light, such as the complexity of equipment installation, obstacles, and the directionality of measurements. Tao et al. (2014) developed an intelligent fruit recognition system that uses feature extraction combined with the nearest neighbor (NN) classifier to achieve fruit and vegetable recognition. The experimental results showed that the recognition rate of the CCLBP (color completed local binary pattern) was 5% higher than that of traditional fruit and vegetable texture feature-detection algorithms. Song et al. (2014) proposed a method for identifying fruits in a greenhouse using a two-step method that combined clustered fruit features. The method has a favorable correlation with manual measurements (94.6%).

Although the analysis method based on a single feature can detect fruits in natural environments, it cannot fully distinguish between target features. Therefore, multi-feature methods are often used to improve robustness and efficiency (Kurtulmus et al., 2011; Rakun et al., 2011; Dey et al., 2012; Yamamoto et al., 2014; Xu et al., 2019).

The use of texture differences, combined with the image color space, geometric features, and other algorithms is more robust for segmenting the target from the background. When the target or fruit is clustered, obstructed or occluded, a histogram was used to separate the color, texture. Moreover, the shape information was used to implement the circular Gabor texture feature, and an intrinsic fruit methodology was used in the fruit recognition algorithm (Zhao et al., 2005; Kurtulmus et al., 2011; Rakun et al., 2011; Xu et al., 2019).

### Multi-Feature Fusion Method

Feature fusion methods combine different features to distinguish different targets. This method can improve the recognition rate of uneven illumination conditions, partially occluded surfaces, and similar background features. These algorithms are widely used for fruit recognition in conjunction with classifiers (Zhuang et al., 2018). A multi-feature integration method for vision systems was proposed to guide fruit-picking robots, clustering multiple feature detection schemes that detect color, morphological, and texture features components of the target region algorithm. The accuracy of multi-feature synthesis reached 90.15% on sunny days and 93.90% on cloudy days (Li et al., 2012). A precise fruit recognition method was proposed based on a set of multiple shared features of multiple robots picking fruits in a mobile environment. The test results showed that the average accuracy of the fruit coding method reached 90% (Kim et al., 2014). Machine vision strategies that combine strength, color, shape, and texture characteristics can also be used to identify fruits (Huang and He, 2012). These four features can be used to train a minimum distance classifier. The resulting experimental images were well-distinguished and had a high likelihood of accurate detection. An artificial neural network with a RIB ratio and textured features was used to segment fruit images; for backlight illuminated image, the segmentation success rate for target in view was above 87.6%, and the bit error rate was about 13% (Zhang et al., 2008). A fruit color recognition method was proposed based on a multi-classifier combination, which combines a support vector machine and fruit color type recognition. The experimental results showed that the average recognition rate was 86.5% (Yang et al., 2016). Algorithms based on a combination of multiple processing and data mining techniques have been proposed to segment fruits in scenes containing different elements and to perform automated harvesting tasks in precision agricultural applications. However, multi-feature fusion methods still prone to light changes, especially in natural environments.

### Deep Learning Method

**FIGURE 6:**
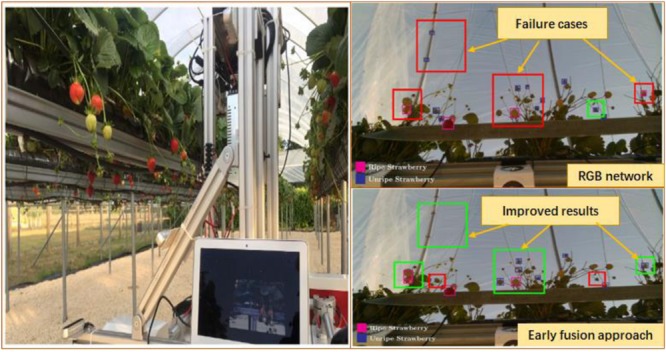
The image acquisition rig inside the strawberry polytunnels and algorithm performance (Kirk et al., 2020) (Improved results are shown in green and detrimental results shown in red).

The concept of deep learning originated from the study of artificial neural networks, which have a multi-layer perceptron with multiple hidden layers. Deep learning can form more abstract high-level attribute categories or features. The high-level features are combined with low-level features to discover distributed feature representations of the data. This technology has been applied in different fields. In fruit recognition, researchers applied deep learning convolutional neural networks (CNN) to the visual techniques of agricultural robots (Yang et al., 2016; Bargoti and Underwood, 2017; Chen et al., 2017; Rahnemoonfar and Sheppard, 2017; Tahir and Badshah, 2018). Hou et al. (2016) developed a fruit recognition algorithm based on a CNN. Fruits and non-fruits were classified using CNN based on image entropy. The trained network produced a significant fruit recognition rate. Fu et al. (2018) studied a faster R-CNN to detect kiwifruit at a recognition rate of 92.3%. Sa et al. (2016) proposed a fruit detection system combined with a deep learning network, using a faster R-CNN model combined with multimodal information (RGB and NIR) for fruit detection. By comparison, this type of model improved the previous model as seen in the test results. Similarly, Horea and Mihai (2018) used deep learning techniques to form a fruit detection data set that was trained using a deep learning model through feature selection; the application software is packaged into the fruit detection system. Horea believed that the system is good at detecting fruit and that more fruit can be added for extensive testing. Kushtrim et al. (2019) proposed that using deep convolution neural network architecture based on single-stage detectors to realize real-time detection of fruits in trees was adopted to improve the detection speed. For video of apples and pears in trees, the detection speed could be increased to 20 frames per second. Compared with the original hard-coded feature extraction algorithms, this method has faster speed. Liu et al. (2018) trained YOLOv3, ResNet50, and ResNet152 deep networks to verify the fruit recognition capability of DNNs. Among them, the best performing ResNet152 network has a recognition accuracy of 95.35% for citrus in natural environments, a recognition accuracy of 97.86% for overlapping citrus fruits, and 85.12% for the leaves and branches of citrus trees. Vitzrabin and Edan (2016) introduced a nine-degree-of-freedom greenhouse sweet pepper harvesting robot. With the help of miniature RGB and ToF cameras, and a 3D point cloud template matching algorithm, fruit positioning was performed; the fruits that were successfully picked accounted for 79% of the total. Kirk et al. (2020) proposed a fast classification and recognition of strawberries by combining color-opponent theory and first-order deep learning network methods (see Figure 6). Its accuracy and recall are 0.793 and 0.799, respectively.

**FIGURE 7:**
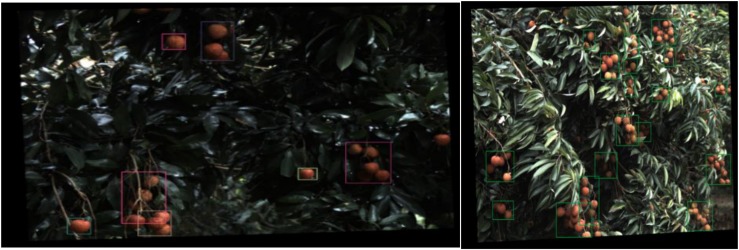
Litchi image recognition effect.

**FIGURE 8:**
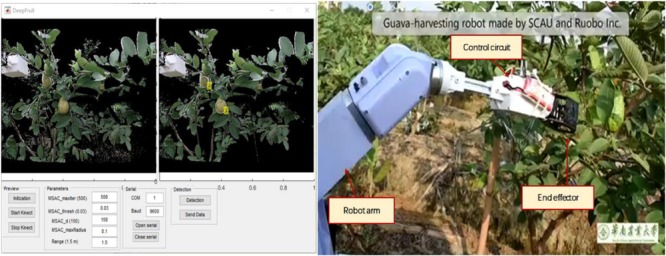
Visual system interface and robot field picking of guava.

To identifying lychee, grape, and other fruits by training the neural network, the authors proposed a more in-depth learning method to divide the fruit image into multiple parts (Luo et al., 2018; Wang et al., 2018; Xiong et al., 2018b), the image features using the Faster R-CNN network algorithm were combined and the lychee and guava fruit were divided into parts and identified. Figure 7 shows the recognition effect of the image of the lychee in the shade and the sun. Based on this algorithm (Lin et al., 2019), a visual picking robot for lightweight guava has been developed shown in Figure 8. Field experiments showed that the accuracy and recall rate of the visual system were 0.88 and 1, respectively. The average image of the vision system required 0.54 s and a robot grab weight (load) of 3.5 kg. The total weight of the 6-degree-of-freedom picking robot is 23 kg. Despite this progress, the operational objects and environments of agricultural harvesting robots are very complex, and their visual algorithms still need to be improved. Despite this progress, the visual algorithms of agriculture robots still need to be improved to deal with the complex operational objects and environments.

A more in-depth learning approach was proposed, which divides the fruit image into multiple parts and use these parts to train the Network combination (Morimoto et al., 2000; Zhang et al., 2008; Gatica et al., 2013; Makkar et al., 2018; Birrell et al., 2019). The purpose of identifying fruits can be achieved with an intensively trained network. Although the deep learning method can produce a higher fruit recognition rate, the model requires a longer training time and is not robust to complicated environments. The training set also requires more image samples. In addition, image classification based on big data has made progress in the crop recognition field, and sometimes small sample image data also exists. Small sample image classification algorithms based on deep learning need to be studied further. This explains why fruit recognition is still a bottleneck despite the prevalent use of deep learning techniques.

### 3D Reconstruction Method for Vision-Based Target

3D reconstruction refers to the establishment of a mathematical model suitable for computer representation and processing of spatial objects. It is the basis for processing, manipulating, and analyzing an object’s properties in a computational environment. In the machine vision of an agricultural robot, 3D reconstruction refers to the process of reversing the 3D information collected about the target by a set of visual sensors. Since the information of each visual image is incomplete, 3D reconstruction often requires the use of empirical knowledge. The purpose of the 3D reconstruction scheme found in fruit-picking robots is multiple: first, to obtain the spatial coordinates of the fruit; second, to guide the robot to the target; third, to determine information such as the posture and shape of the fruit; fourth, to provide information that the robot end-effector can use for to establish a behavioral decision. The 3D reconstruction process based on a visual image is as follows:

1. Camera calibration: The camera calibration is done to establish an imaging model, to ensure the internal and external parameters of the camera are resolved and ensure that the coordinates of the image can be combined to obtain the coordinates of multiple 3D points in the space.
2. Image acquisition: Before performing image processing, the camera is used to acquire a two-dimensional image of a 3D object.
3. Feature extraction: Features mainly include feature points, lines, and regions. In most cases, the feature points are used as matching primitives, the form in which the feature points are extracted is closely related to the matching strategy.
4. Stereo matching: Stereo matching refers to the correspondence between image pairs according to their extracted features, that is, one-to-one correspondence of imaging points of the same physical space point in two different images.
5. 3D reconstruction: After stereo matching, the 3D scene information can be recovered using the internal and external parameters of the camera calibration.

Over the years, researchers have carried out extensive research on the orchard environment, the visual recognition, and the 3D positioning of the objects picked.

Keerthy et al. (2017) used RGB-D sensors to perform 3D positioning for automatic broccoli harvesting. Mature broccoli heads in the field were detected. They evaluated the application of different 3D features, machine learning and time filtering methods in broccoli head detection. The recognition results and the point cloud showed that the 3D position of the broccoli was accurate.

To minimize the impact of outdoor changes in illumination on the visual 3D reconstruction of the image, Gongal et al. (2016) developed a new vision sensor system that used a cross-platform for flushing the apple tree and images were obtained from both sides of the apple tree. The platform structure protects the apple tree from direct sunlight, significantly reducing the illumination changes and increasing the recognition rate of the apple. Luo et al. (2016) determined the anti-collision space surrounding of grape-based on binocular stereo vision and reconstructed the spatial encirclement of the grape cluster by solving the spatial coordinates of each grapefruit. This provided the decision for robot anti-collision picking behavior. Ehud et al. (2016) proposed an object pose solution method for RGB images and shape features of objects in 3D space. This method combines 3D surface normal features, 3D plane reflection symmetry, and image plane highlights from the elliptical surface points to detect fruit. After using the proposed algorithm, the mean average precision improved (from 0.52 to 0.55). Onishi et al. (2019) used a multi-box detector and a stereo camera to detect the 3D position of the target fruit, and then controlled the robotic arm to harvest the fruit by rotating the hand shaft. More than 90% of the fruit targets were detected, and the single fruit harvest time was about 16 s.

**FIGURE 9:**
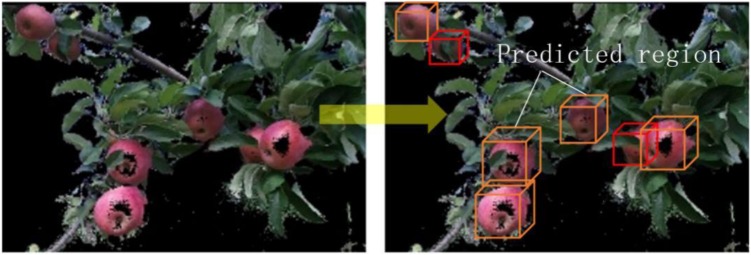
Apple identification implemented via RGB-D camera and SVM classifier (Tao and Zhou, 2017).

To accurately identify and locate litchi fruits under non-structural dynamic environment, Xiong et al. (2018b) proposed a method for calculating the location of disturbed litchi picking points based on binocular stereo vision motion analysis. This method uses the principle of single pendulum motion to establish the vibration angle of litchi clusters under static, small disturbance and large disturbance. Improved fuzzy C-means clustering method was used to segment the litchi fruits and stem segments. The binocular stereo vision was also used. The picking point space coordinates were calculated. Williams H. A. M. et al. (2019) designed a kiwi fruit picking visual system by combining deep neural network and stereoscopic vision technology, which can reliably detect kiwi fruit under natural light. With the new end-effect-designed system, 51% kiwi fruit in the orchard can be picked, and the average time spent on picking a kiwi fruit is only 5.5 s. To facilitate the picking robot to carry out mobile navigation operations in the orchard, Matsuzaki et al. (2018) obtained orchard point cloud data to reconstruct 3D scenes and provide support for mobile navigation path planning of fruit-picking robots. This research has revealed the important role of mapping methods in agricultural robots, which can provide robots with more comprehensive environmental information. On the other hand, depth monitoring cameras are well integrated into agricultural mapping tasks. This work gives a good example of integrating different modules such as deep learning, depth cameras and mapping into agricultural tasks. Lee et al. (2019) used three cameras to develop the visual servo system of sweet pepper automatic harvest. Two cameras were used to achieve stereoscopic vision, and the third camera was used to act together with the end-effector to correct the pose of sweet pepper during the action. To improve the recognition and perception of the apple picking robot in 3D space, Tao and Zhou (2017) proposed an automatic apple recognition method based on point cloud data, which first acquired the orchard point cloud data via the RGB-D camera of time of flight technology, and merged the color features. 3D geometric features were extracted from point cloud data. The point cloud data is further divided into apples, branches, and leaves to provide a more comprehensive sensing capability for the system. The three data classifiers are optimized by using support vector machine and genetic algorithm. Finally, the accurate recognition of the fruit target is achieved (see Figure 9). The classification accuracy rates obtained by the proposed Color-FPFH features for apples, branches and leaves are 92.30, 88.03, and 80.34% respectively, which are significantly higher than those of the compared features from different algorithms.

During the process of harvesting crops, in addition to environmental noise, the surrounding background related to positioning also contains branches and leaves. Because the fruit grows on the fruit branches when it is positioned, it will cause interference and caused the collide between the branches and leaves, lending it inaccurate positioning. Therefore, these branches and leaves are treated as an obstacle, and an obstacle map is used to describe 3D spatial information. Decision-making behavior was used by picking robots to avoid obstacles. Bac et al. (2014a) used a support line wrapped around a pepper stem as a visual clue to detect pepper stems. The correct rate of this method was 0.94. Zhang and Xu (2018) proposed an unsupervised conditional random field algorithm to cluster tomato plants with leaves and stems. Majeed et al. (2018) segmented the apple tree trunks and branches in the RGB-D image and achieved favorable results.

Through the comparison and analysis of the literature on 3D reconstruction of the picking robot, it is found that current 3D reconstruction methods require a binocular stereo vision system (Si et al., 2015; Wang et al., 2016) or an RGB-D based vision system (Wang et al., 2016; Qureshi et al., 2017; Sa et al., 2017; Matsuzaki et al., 2018; Lin et al., 2019). Among the algorithms, an algorithm based on binocular stereoscopic 3D reconstruction mainly focuses on visual stereo matching and fruit feature point extraction in complex orchard environments. 3D reconstruction based on the RGB-D vision sensor mainly focused on point cloud data processing and target extraction. The posture reversal of the picking target is the output of the algorithm.

Unmanned aerial vehicles-based 3D technology has been widely applied to agriculture due to its unique advantages such as crop yield evaluation and disease monitoring (Honkavaara et al., 2012; Chen et al., 2019; Vanbrabant et al., 2019). Additional research can be found in Sekhar et al. (2010) and Comba et al. (2018). Although the technology provided by this literature is not directly related to fruit picking robots, this literature provides information about the advancement in technology for fruit recognition. We have not retrieved literature about UAV-based fruit picking robots. However, in some patents, the inventors proposed the design scheme of fruit picking robots mounted on UAVs, and some companies also published information about UAV-based fruit picking robotics on their home pages. Unfortunately, specific flight picking technology could be not investigated and may be investigated in future research.

## Visual Fault Tolerance

The working environment for agricultural picking robots is complex. There are often many disturbances and occlusions. Thus, the robotic visual positioning system often encounters large random errors, and the laws of these errors are difficult to describe and compensate. For this reason, the collaboration between robot vision and mechanical fault tolerance has become a research hotspot in recent years. For significant random errors, traditional vision does not consider mechanical or visual correlations; Fruits were found by acquiring 3D coordinates. The original system can only compensate for the original error caused by the visual hardware. Thus, it is difficult to compensate for unknown random errors. Second, the calculation error and transitivity need to be applied because the image processing and calculation separate the background and the fruit trees in the scene, and then processes the fruit trees, branches, or leaves. These errors are found using the part of the processing and calculation process of multi-objective image. Calculation errors can also be caused by noise. Errors, especially their transitivity, are easily overlooked. Therefore, visual and institutional synergy is needed to correct various types of errors. The main influencing factors need to be further studied such as calculation error, random error, and transitivity.

Robot positioning errors and fault tolerance have attracted the attention of scholars in various fields (Gong et al., 2019; Guo et al., 2019; Rasouli et al., 2020). Blas and Blanke (2011) have studied automatic baling robots, which can locate plants using binocular vision and classify the appearance of plants. Finally, the fault tolerance of the machine was also verified. Zou et al. (2016) proposed the concept of mechanical and visual coordination fault tolerance. By analyzing the spatial distribution range and peak characteristics of random errors and finding out random variables and transmitting the main factors, the factors that influence the error with a high probability were separated, and an evaluation model was constructed. The comprehensive error compensation model is used for data organization, vision, and control (Zou et al., 2016). Xiong et al. (2019) developed a strawberry harvesting robot with a cable-driven gripper. An RGB-D camera was used to detect strawberries. A fixture embedded with an infrared sensor was designed to compensate for positioning errors in the vision system, which has good stability (Xiong et al., 2019). In the field of plant harvesting robots, the fault tolerance theory needs to be further explored in order to achieve precise operation and improve the reliability of the machine.

## Future Perspective

This paper reviewed stereo vision technology and the application of harvesting robots. Research into stereo vision systems mainly included crop identification and localization, stereo vision and cooperative behavior control of the robot manipulator, and error processing. The system identifies the crop by collecting a set of images and extracting information about the target. To identify fruit, the resulting fruit, fruit branch and the fruit branch obstacle in a 3D reconstruction are processed via space coordinate calculation. After this calculation is complete, the calculated spatial coordinates are transmitted to the robot drive. The system controls the robot’s work. Algorithms and intelligent decision-making are implemented through visual software.

When the crop environment is different under the illumination and occlusion conditions of the field environment, the recognition and location accuracy are affected. Geometric features, image features, new image algorithms, and intelligent decision theory was applied by the researchers to solve the problem. In most image algorithms, current deep learning algorithms require a large number of samples. In agricultural crops, sometimes only a small dataset can be obtained, such as immature fruits and crops with a pest problem. The deep learning image recognition methods that process these small datasets needs further research.

Although artificial intelligence and its deep learning methods have improved the recognition rate, there are still large positioning errors in the application of the visual system due to the complexity and uncertainty of the agricultural environment. Thus, incorporating the robot’s control system and the innovative design of the mechanism is required to improve the vision and combined error tolerance technology, to achieve precise positioning and operation. Further researches are also worth exploring for combining artificial intelligence technology with the robot’s active fault tolerance and its intelligent behavior decision.

Agricultural harvesting robots are subject to dynamic interference from external forces during operation. The key technology is dynamic tracking of curved surfaces from irregularly shaped fruits. The dynamic tracking of the object with high accuracy remains an unsolved issue for visual harvesting robots.

## Author Contributions

YT and MC provided conceptualization and writing. CW and LL provided writing and validation. JL and GL provided writing and investigation. XZ provided and supervision.

## Conflict of Interest

The authors declare that the research was conducted in the absence of any commercial or financial relationships that could be construed as a potential conflict of interest.

## References

[B1] Arad B. Balendonck J. Barth R. Ben-Shahar O. Edan Y. Hellström T. (2020). Development of a sweet pepper harvesting robot. *J. Field Robot.* 1–13. 10.1002/rob.21937 27527168

[B2] Arad B. Kurtser P. Barnea E. Harel B. Edan Y. Ben-Shahar O. (2019). Controlled Lighting and Illumination-Independent Target Detection for Real-Time Cost-Efficient Applications. The Case Study of Sweet Pepper Robotic Harvesting. *Sensors* 19 1–15. 10.3390/s19061390 30901837 PMC6470490

[B3] Arefi A. Motlagh A. M. Mollazade K. Teimourlou R. F. (2011). Recognition and localization of ripen tomato based on machine vision. *Aust. J. Crop Sci.* 5 1144–1149.

[B4] Bac C. W. Hemming J. van Henten E. J. (2014a). Stem localization of sweet-pepper plants using the support wire as a visual cue. *Comput. Electr. Agricult.* 105 111–120.

[B5] Bac C. W. Henten E. J. Hemming J. Edan Y. (2014b). Harvesting robots for high-value crops: state-of-the-art review and challenges ahead. *J. Field Robot.* 31:888e911.

[B6] Bargoti S. Underwood J. P. (2017). Image Segmentation for Fruit Detection and Yield Estimation in Apple Orchards. *J. Field Robot.* 34 1039–1060.

[B7] Barth R. Hemming J. Van Henten E. J. (2019). Angle estimation between plant parts for grasp optimisation in harvest robots. *Biosyst. Eng.* 183 26–46.

[B8] Bechar A. (2010). Robotics in horticultural field production. *Stewart Postharvest Rev.* 6 1–11. 10.1111/plb.12914 30230154

[B9] Birrell S. Hughes J. Cai J. Y. Iida F. (2019). A field-tested robotic harvesting system for iceberg lettuce. *J. Field Robot.* 37 1–21. 10.1002/rob.21888 32194355 PMC7074041

[B10] Blas M. R. Blanke M. (2011). Stereo vision with texture learning for fault-tolerant automatic baling. *Comput. Electr. Agricul.* 75 159–168.

[B11] Blok P. M. van Boheemen K. van Evert F. K. IJsselmuiden J. Kim G. (2019). Robot navigation in orchards with localization based on Particle filter and Kalman filter. *Comput. Electr. Agricult.* 157 261–269.

[B12] Brown G. K. Schertz C. E. (1967). Evaluating Shake Harvesting of Oranges for the Fresh Fruit Market. *Trans. ASAE* 10 577–578.

[B13] Buemi F. Massa M. Sandini G. Costi G. (1996). The AGROBOT project. *Adv. Space Res.* 18 185–189. 11538962 10.1016/0273-1177(95)00807-q

[B14] Bulanon D. M. Burks T. F. Alchanatis V. (2010). A multispectral imaging analysis for enhancing citrus fruit detection. *Environ. Control Biol.* 48 81–91.

[B15] Bulanon D. M. Kataoka T. Okamoto H. Hata S. (2004). “Development of a real-time machine vision system for the apple harvesting robot,” in *Proceedings of the SICE Annual Conference in Sapporo, Hokkaido Institute of Technolgy*, Japan, 595–598.

[B16] Bulanon D. M. Kataoka T. Ota Y. Hiroma T. (2002). AE—automation and emerging technologies: a segmentation algorithm for the automatic recognition of Fuji apples at harvest. *Biosyst. Eng.* 83 405–412.

[B17] Ceres R. Pons J. L. Jiménez A. R. Martín J. M. Calderón L. (1998). Design and implementation of an aided fruit-harvesting robot (Agribot). *Indus. Robot* 25 337–346.

[B18] Chen S. W. Shivakumar S. S. Dcunha S. Das J. Okon E. Qu C. (2017). Counting apples and oranges with deep learning: a data-driven approach. *IEEE Robot. Automat. Lett.* 2 781–788.

[B19] Chen Y. Lee W. S. Gan H. Peres N. Fraisse C. Zhang Y. (2019). Strawberry yield prediction based on a deep neural network using high-resolution aerial orthoimages. *Remote Sens.* 11:1584.

[B20] Comba L. Biglia A. Aimonino D. R. Gay P. (2018). Unsupervised detection of vineyards by 3D point-cloud UAV photogrammetry for precision agriculture. *Comput. Electr. Agricult.* 155 84–95.

[B21] Cubero S. Aleixos N. Albert F. Torregrosa A. Ortiz C. García-Navarrete O. (2014). Optimised computer vision system for automatic pre-grading of citrus fruit in the field using a mobile platform. *Precision Agricult.* 15 80–94.

[B22] Daudelin J. Jing G. Tosun T. Yim M. Kress-Gazit H. Campbell M. (2018). An integrated system for perception-driven autonomy with modular robots. *Sci. Robot.* 3 1–6. 10.1126/scirobotics.aat4983 33141737

[B23] Dey D. Mummert L. Sukthankar R. (2012). “Classification of plant structures from uncalibrated image sequences,” in *Proceedings of the 2012 IEEE Workshop on the Applications of Computer Vision (WACV)*, Breckenridge, CO, 329–336.

[B24] d’Grand E. Rabatel A. G. Pellenc R. Journeau A. Aldon M. J. (1987). Magali: A self-propelled robot to pick apples. *Am. Soc. Agricult. Eng. Paper* 46 353–358.

[B25] Edan Y. Han S. Kondo N. (2009). *Automation in Agriculture, Springer Handbook of Automation.* Berlin: Springer Berlin Heidelberg, 1095–1128.

[B26] Edan Y. Rogozin D. Flash T. Miles G. E. (2000). Robotic melon harvesting. *IEEE Trans. Robot. Automat.* 16 831–835. 19839306

[B27] Ehud B. Rotem M. Ohad B. (2016). Colour-agnostic shape-based 3D fruit detection for crop harvesting robots. *Biosyst. Eng.* 146 57–70.

[B28] Fischer J. W. Greiner K. Lutman M. W. Webber B. L. Vercauteren K. C. (2019). Use of unmanned aircraft systems (UAS) and multispectral imagery for quantifying agricultural areas damaged by wild pigs. *Crop Protect.* 125:104865.

[B29] Fu L. Feng Y. Majeed Y. Zhang X. Zhang J. Karkee M. (2018). Kiwifruit detection in field images using Faster R-CNN with ZFNet. *IFAC Papers On Line* 51 45–50.

[B30] Fu L. Tola E. Al-Mallahi A. Li R. Cui Y. (2019). A novel image processing algorithm to separate linearly clustered kiwifruits. *Biosyst. Eng.* 183 184–195.

[B31] Garcia-Sanchez A. Garcia-Sanchez F. Garcia-Haro J. (2011). Wireless sensor network deployment for integrating video-surveillance and data-monitoring in precision agriculture over distributed crops. *Comput. Electr. Agricult.* 75 288–303.

[B32] Gatica G. Best S. Ceroni J. Lefranc G. (2013). Olive Fruits Recognition Using Neural Networks. *Proc. Comput. Sci.* 17 412–419.

[B33] Gong J. Y. Jiang B. Shen Q. K. (2019). Adaptive fault-tolerant neural control for large-scale systems with actuator faults. *Int. J. Control Automat. Syst.* 17 1421–1431. 10.1109/TNNLS.2016.2598580 27552769

[B34] Gongal A. Amatya S. Karkee M. Zhang Q. Lewis K. (2015). Sensors and systems for fruit detection and localization: a review. *Comput. Electr. Agricult.* 116 8–19.

[B35] Gongal A. Silwal A. Amatya S. Karkee M. Zhang Q. Lewis K. (2016). Apple crop-load estimation with over-the-row machine vision system. *Comput. Electr. Agricult.* 120 26–35.

[B36] Grift T. Zhang Q. Kondo N. Ting K. C. (2008). A review of automation and robotics for the bio- industry. *J. Biomechatr. Eng.* 1 37–54.

[B37] Guo C. Li F. Tian Z. Guo W. Tan S. (2019). Intelligent active fault-tolerant system for multi-source integrated navigation system based on deep neural network. *Neural Comput. Appl.* 1 1–18.

[B38] Hannan M. W. Burks T. F. Bulanon D. M. (2007). A real-time machine vision algorithm for robotic citrus harvesting. *Trans. ASABE* 8 1–11.

[B39] Hannan M. W. Burks T. F. Bulanon D. M. (2009). A machine vision algorithm combining adaptive segmentation and shape analysis for orange fruit detection. *CIGR J.* 6 1–17.

[B40] Harrell R. C. Slaughter D. C. Adsit P. D. (1985). Vision guidance of a robotic tree fruit harvester. *Intellig. Robot. Comput. Vis.* 579 537–545.

[B41] Hayashi S. Ganno K. Ishii Y. Tanaka I. (2002). Robotic harvesting system for eggplants. *JARQ Jpn. Agricult. Res. Q.* 36 163–168.

[B42] Hayashi S. Ota T. Kubota K. Ganno K. Kondo N. (2005). Robotic harvesting technology for fruit vegetables in protected horticultural production. *Symp. Édn. Q.* 5 227–236.

[B43] Hemming J. Ruizendaal J. Hofstee J. W. van Henten E. J. (2014). Fruit detectability analysis for different camera positions in sweet-pepper. *Sensors* 14 6032–6044. 10.3390/s140406032 24681670 PMC4029692

[B44] Hiroaki M. Jun M. Shuji O. (2017). “Development of a mobile robot for harvest support in greenhouse horticulture - Person following and mapping,” in *Proceedings of the 2017 IEEE/SICE International Symposium on System Integration (SII)*, Taipei, 541–546.

[B45] Honkavaara E. Kaivosoja J. Mäkynen J. Pellikka I. Pesonen L. Saari H. (2012). Hyperspectral reflectance signatures and point clouds for precision agriculture by light weight UAV imaging system. *ISPRS Ann. Photogramm. Remote Sens. Spat. Inf. Sci.* 7 353–358.

[B46] Horea M. Mihai O. (2018). Fruit recognition from images using deep learning. *Acta Univ. Sapientiae Inform.* 10 26–42. 10.3389/fpls.2019.00611 31178875 PMC6537632

[B47] Hou L. Wu Q. Sun Q. Yang H. Li P. (2016). “Fruit recognition based on convolution neural network,” in *Proceedings of the 2016 12th International Conference on Natural Computation, Fuzzy Systems and Knowledge Discovery (ICNC-FSKD)*, Changsha, 18–22.

[B48] Huang L. He D. (2012). Ripe fuji apple detection model analysis in natural tree canopy. *Telkomnika Indonesian J. Electr. Eng.* 10 1771–1778.

[B49] Hung C. Nieto J. Taylor Z. Underwood J. Sukkarieh S. (2013). “Orchard fruit segmentation using multi-spectral feature learning,” in *Proceedings of the 2013 IEEE/RSJ International Conference on Intelligent Robots and Systems*, Piscataway, NJ, 5314–5320.

[B50] Jimenez A. R. Ceres R. Pons J. L. (1999). “A machine vision system using a laser radar applied to robotic fruit harvesting,” in *Proceedings of the IEEE Workshop on Computer Vision Beyond the Visible Spectrum: Methods and Applications (CVBVS’*99), Hilton Head, SC, 110–119.

[B51] Jiménez A. R. Ceres R. Pons J. L. (2000a). A survey of computer vision methods for locating fruit on trees. *Trans. ASAE* 43 1911–1920.

[B52] Jiménez A. R. Ceres R. Pons J. L. (2000b). A vision system based on a laser range-finder applied to robotic fruit harvesting. *Mach. Vis. Appl.* 11 321–329.

[B53] Zhao Y. Gong L. Huang Y. Liu C. (2016). Robust tomato recognition for robotic harvesting using feature images fusion. *Sensors* 16:173. 10.3390/s16020173 26840313 PMC4801551

[B54] Kapach K. Barnea E. Mairon R. Edan Y. Ben-Shahar O. (2012). Computer vision for fruit harvesting robots - state of the art and challenges ahead. *Int. J. Comput. Vis. Robot.* 3 4–34.

[B55] Keerthy K. Tomáš K. Simon P. Tom D. Grzegorz C. (2017). 3D-vision based detection, localization, and sizing of broccoli heads in the field. *J. Field Robot.* 34 1505–1518.

[B56] Kendo N. Nishitsuji Y. Ling P. P. Ting K. C. (1996). Visual feedback guided robotic cherry tomato harvesting. *Am. Soc. Agricult. Eng.* 39 2331–2338.

[B57] Khaliq A. Comba L. Biglia A. Ricauda Aimonino D. Chiaberge M. Gay P. (2019). Comparison of satellite and UAV-based multispectral imagery for vineyard variability assessment. *Remote Sens.* 11:436.

[B58] Kim J. Vogl M. Kim S. (2014). “A code based fruit recognition method via image convertion using multiple features,” in *Proceedings of the 2014 International Conference on IT Convergence and Security (ICITCS)*, Beijing, 1–4.

[B59] Kirk R. Cielniak G. Mangan M. (2020). L^∗^a^∗^b^∗^Fruits: a rapid and robust outdoor fruit detection system combining bio-inspired features with one-stage deep learning networks. *Sensors* 20:275. 10.3390/s20010275 31947829 PMC6983004

[B60] Kitamura S. Oka K. (2005). “Recognition and cutting system of sweet pepper for picking robot in greenhouse horticulture,” in *Proceedings of the IEEE International Conference Mechatronics and Automation*, Beijing, 1807–1812.

[B61] Kondo N. Shunzo E. (1989). Methods of detecting fruit by visual sensor attached to manipulator. *J. Jpn. Soc. Agricult. Mach.* 51 41–48.

[B62] Kondo N. Ting K. C. (1998). Robotics for plant production. *Artif. Intellig. Rev.* 12 227–243.

[B63] Kondo N. Yamamoto K. Shimizu H. Yata K. Kurita M. Shiigi T. (2009). A machine vision system for tomato cluster harvesting robot. *Eng. Agricult. Environ. Food* 2 60–65.

[B64] Kong D. Zhao D. Zhang Y. Wang J. Zhang H. (2010). “Research of apple harvesting robot based on least square support vector machine,” in *Proceedings of the 2010 International Conference on Electrical and Control Engineering*, Washington, DC, 1590–1593.

[B65] Kurtulmus F. Lee W. S. Vardar A. (2011). Green citrus detection using ‘eigenfruit’, color and circular Gabor texture features under natural outdoor conditions. *Comput. Electr. Agricult.* 78 140–149.

[B66] Kushtrim B. Demetrio P. G. Alexandra B. Brunella M. Corelli G. L. Luigi M. (2019). Single-shot convolution neural networks for real-time fruit detection within the tree. *Front. Plant Sci.* 10:611. 10.3389/fpls.2019.00611 31178875 PMC6537632

[B67] Lee B. Kam D. Min B. Hwa J. Oh S. (2019). A vision servo system for automated harvest of sweet pepper in Korean greenhouse environment. *Appl. Sci.* 9:2395.

[B68] Li B. Vigneault C. Wang N. (2010). Research development of fruit and vegetable harvesting robots in China. *Stewart Postharvest Rev.* 6 1–8.

[B69] Li J. de Avila B. E. Gao W. Zhang L. Wang J. (2017). Micro/nanorobots for biomedicine: delivery, surgery, sensing, and detoxification. *Sci. Robot.* 2 1–9. 10.1126/scirobotics.aam6431 31552379 PMC6759331

[B70] Li M. Imou K. Wakabayashi K. Yokoyama S. (2009). Review of research on agricultural vehicle autonomous guidance. *Int. J. Agricult. Biol. Eng.* 2 1–26.

[B71] Li P. Lee S. Hsu H. (2011). “Study on citrus fruit image using fisher linear discriminant analysis,” in *Proceedings of the 2011 IEEE International Conference on Computer Science and Automation Engineering*, Shanghai, 175–180.

[B72] Li X. Li L. Gao Z. Zhou J. Min S. (2012). Image recognition of camellia fruit based on preference for aiNET multi-features integration. *Trans. Chin. Soc. Agricult. Eng.* 28 133–137.

[B73] Lin G. Tang Y. Zou X. Xiong J. Li J. (2019). Guava detection and pose estimation using a low-cost RGB-D sensor in the field. *Sensors* 19 1–15. 10.3390/s19020428 30669645 PMC6359182

[B74] Liu T. Ehsani R. Toudeshki A. Zou X. Wang H. (2019). Identifying immature and mature pomelo fruits in trees by elliptical model fitting in the Cr-Cb color space. *Precis. Agricult.* 20 138–156.

[B75] Liu Y. P. Yang C. H. Ling H. Mabu S. Kuremoto T. (2018). “A visual system of citrus picking robot using convolutional neural networks,” in *Proceedings of the 2018 5th International Conference on Systems and Informatics (ICSAI)*, Nanjing, 344–349.

[B76] Liu Z. Liu G. (2007). Apple maturity discrimination and positioning system in an apple harvesting robot. *New Zealand J. Agricult. Res.* 50 1103–1113.

[B77] Lu Q. Tang M. Cai J. (2011). “Obstacle recognition using multi-spectral imaging for citrus picking robot,” in *Proceedings of the 2011 Third Pacific-Asia Conference on Circuits, Communications and System (PACCS)*, Wuhan, 1–5.

[B78] Luo L. Tang Y. Lu Q. Chen X. Zhang P. Zou X. (2018). A vision methodology for harvesting robot to detect cutting points on peduncles of double overlapping grape clusters in a vineyard. *Comput. Indus.* 99 130–139.

[B79] Luo L. Tang Y. Zou X. Ye M. Feng W. Li G. (2016). Vision-based extraction of spatial information in grape clusters for harvesting robots. *Biosyst. Eng.* 151 90–104.

[B80] Majeed Y. Zhang J. Zhang X. Fu L. Karkee M. Zhang Q. (2018). Apple tree trunk and branch segmentation for automatic trellis training using convolutional neural network based semantic segmentation. *IFAC PapersOnLine* 51 75–80.

[B81] Makkar T. Verma S. Yogesh A. Dubey A. K. (2018). “Analysis and detection of fruit defect using neural network,” in *Data Science and Analytics. REDSET 2017. Communications in Computer and Information Science*, eds Panda B. Sharma S. Roy N. (Singapore: Springer), Vol 799 554–567.

[B82] Makky M. Soni P. (2013). Development of an automatic grading machine for oil palm fresh fruits bunches (FFBs) based on machine vision. *Comput. Electr. Agricult.* 93 129–139.

[B83] Matsuzaki S. Masuzawa H. Miura J. Oishi S. (2018). “3D semantic mapping in greenhouses for agricultural mobile robots with robust object recognition using robots’ trajectory,” in *Proceedings of the 2018 IEEE International Conference on Systems, Man, and Cybernetics (SMC)*, Toronto, 357–362.

[B84] Mehta S. S. MacKunis W. Burks T. F. (2014). Nonlinear robust visual servo control for robotic citrus harvesting. *IFAC Proc. Vol.* 47 8110–8115.

[B85] Morimoto T. Takeuchi T. Miyata H. Hashimoto Y. (2000). Pattern recognition of fruit shape based on the concept of chaos and neural networks. *Comput. Electr. Agricult.* 26 171–186.

[B86] Navia J. Mondragon I. Patino D. Colorado J. (2016). “Multispectral mapping in agriculture: terrain mosaic using an autonomous quadcopter UAV,” in *Proceedings of the 2016 International Conference on Unmanned Aircraft Systems (ICUAS)*, Kaisariani, 1351–1358.

[B87] Onishi Y. Yoshida T. Kurita H. Fukao T. Arihara H. Iwai A. (2019). An automated fruit harvesting robot by using deep learning. *ROBOMECH J.* 6:13. 10.3390/s19204599 31652634 PMC6832306

[B88] Peng H. Zou X. Chen L. Xiong J. Chen K. Lin G. (2014). Fast recognition of multiple color targets of litchi image in field environment based on Double Otsu algorithm. *Trans. Chin. Soc. Agricult. Eng.* 45 61–68.

[B89] Plá F. Juste F. Ferri F. (1993). Feature extraction of spherical objects in image analysis: an application to robotic citrus harvesting. *Comput. Electr. Agricult.* 8 57–72.

[B90] Plebe A. Grasso G. (2001). Localization of spherical fruits for robotic harvesting. *Mach. Vis. Appl.* 13 70–79.

[B91] Qingchun F. Xiu W. Wengang Z. Quan Q. Kai J. (2012). New strawberry harvesting robot for elevated-trough culture. *Int. J. Agricult. Biol. Eng.* 5 1–8.

[B92] Qureshi W. Payne A. Walsh K. Linker R. Cohen O. Dailey M. (2017). Machine vision for counting fruit on mango tree canopies. *Precision Agricult.* 18 224–244. 10.3390/s19122742 31216769 PMC6631562

[B93] Rahnemoonfar M. Sheppard C. (2017). Deep count: fruit counting based on deep simulated learning. *Sensors* 17:9054. 10.3390/s17040905 28425947 PMC5426829

[B94] Rakun J. Stajnko D. Zazula D. (2011). Detecting fruits in natural scenes by using spatial-frequency based texture analysis and multiview geometry. *Comput. Electr. Agricult.* 76 80–88.

[B95] Rasouli P. Forouzantabar A. Moattari M. Azadi M. (2020). Fault-tolerant control of teleoperation systems with flexible-link slave robot and disturbance compensation. *Irani. J. Sci. Technol. Trans. Electr. Eng.* 1–13. 10.1007/s40998-020-00309-5

[B96] Reis M. J. C. S. Morais R. Peres E. Pereira C. Contente O. Soares S. (2012). Automatic detection of bunches of grapes in natural environment from color images. *J. Appl. Logic* 10 285–290.

[B97] Roberts L. (1965). Machine perception of three-dimension solids, in optical and electro-optimal. *Form. Process.* 10 190–193.

[B98] Sa I. Ge Z. Dayoub F. Upcroft B. Perez T. McCool C. (2016). DeepFruits: a fruit detection system using deep neural networks. *Sensors* 16:12228. 10.3390/s16081222 27527168 PMC5017387

[B99] Sa I. Lehnert C. English A. McCool C. Dayoub F. Upcroft B. (2017). Peduncle Detection of Sweet Pepper for Autonomous Crop Harvesting - Combined Colour and 3D Information. *IEEE Robot. Automat. Lett.* 2 765–772.

[B100] Sarig Y. (1993). Robotics of fruit harvesting: a state-of-the-art review. *J. Agricult. Eng. Res.* 54 265–280.

[B101] Scarfe A. J. Flemmer R. C. Bakker H. H. Flemmer C. L. (2009). “Development of an autonomous kiwifruit picking robot,” in *Proceedings of the 4th International Conference on Autonomous Robots and Agents*, Wellington, 639–643.

[B102] Sekhar P. S. Gerrit H. Paz J. O. (2010). Remote sensing and geospatial technological applications for site-specific management of fruit and nut crops: a review. *Remote Sens.* 2 1973–1997.

[B103] Si Y. Liu G. Feng J. (2015). Location of apples in trees using stereoscopic vision. *Comput. Electr. Agricult.* 112 68–74.

[B104] Silwal A. Davidson J. Karkee M. Mo C. Zhang Q. Lewis K. (2016). “Effort towards robotic apple harvesting in Washington State,” in *Proceedings of the 2016 ASABE Annual International Meeting. American Society of Agricultural and Biological Engineers*, Boston, MA, 1–11.

[B105] Silwal A. Davidson J. R. Karkee M. Mo C. Zhang Q. Lewis K. (2017). Design, integration, and field evaluation of a robotic apple harvester. *J. Field Robot.* 34 1140–1159.

[B106] Slaughter D. Harrell R. Adsit P. (1986). Image enhancement in robotic fruit harvesting. *Am. Soc. Agricult. Eng. Microfiche Collect.*

[B107] Slaughter D. C. Harrell R. C. (1987). Color Vision in Robotic Fruit Harvesting. *Trans. ASAE* 30 1144–1148.

[B108] Song Y. Glasbey C. A. Horgan G. W. Polder G. Dieleman J. A. van der Heijden G. W. A. M. (2014). Automatic fruit recognition and counting from multiple images. *Biosyst. Eng.* 118 203–215.

[B109] Tahir M. Badshah S. (2018). Extracting accurate time domain features from vibration signals for reliable classification of bearing faults. *Adv. Appl. Sci.* 5 156–163.

[B110] Takahashi T. Zhang S. H. Fukuchi H. Bekki E. (2000). Binocular stereo vision system for measuring distance of apples in orchard, 2: analysis of and solution to the correspondence problem. *J. Jpn. Soc. Agricult. Mach.* 62 88–89.

[B111] Tang Y. Li L. Feng W. Liu F. Zou X. Chen M. (2018). Binocular vision measurement and its application in full-field convex deformation of concrete-filled steel tubular columns. *Measurement* 130 372–383.

[B112] Tanigaki K. Fujiura T. Akase A. Imagawa J. (2008). Cherry-harvesting robot. *Comput. Electr. Agricult*, 63 65–72.

[B113] Tao H. Zhao L. Xi J. Yu L. Wang T. (2014). Fruits and vegetables recognition based on color and texture features fruits and vegetables recognition based on color and texture features. *Trans. Chin. Soc. Agricult. Eng.* 30 305–311.

[B114] Tao Y. Zhou J. (2017). Automatic apple recognition based on the fusion of color, and 3D feature for robotic fruit picking. *Comput. Electr. Agricult.* 142 388–396.

[B115] Van Henten E. J. (2006). “Greenhouse mechanization: state of the art and future perspective,” in *Proceedings of the Acta Horticulturae*, Cameron Highlands, 55–69.

[B116] Van Henten E. J. Van T. Slot D. A. Hol C. W. J. Van Willigenburg L. G. (2009). Optimal manipulator design for a cucumber harvesting robot. *Comput. Electr. Agricult.* 65 247–257.

[B117] Van Henten E. J. Van Tuijl B. A. J. Hemming J. Kornet J. G. Bontsema J. Van Os E. A. (2003). Field test of an autonomous cucumber picking robot. *Biosyst. Eng.* 86 305–313.

[B118] Vanbrabant Y. Tits L. Delalieux S. Pauly K. Verjans W. Somers B. (2019). Multitemporal Chlorophyll mapping in pome fruit orchards from remotely piloted aircraft systems. *Remote Sens.* 11:1468.

[B119] Vitzrabin E. Edan Y. (2016). Changing task objectives for improved sweet pepper detection for robotic harvesting. *IEEE Robot. Automat. Lett.* 1 578–584.

[B120] Wang C. Lee W. S. Zou X. Choi D. Gan H. Diamond J. (2018). Correction to: Detection and counting of immature green citrus fruit based on the Local Binary Patterns (LBP) feature using illumination-normalized images. *Precision Agricult.* 19:1084.

[B121] Wang C. Tang Y. Zou X. SiTu W. Feng W. (2017). A robust fruit image segmentation algorithm against varying illumination for vision system of fruit harvesting robot. *OPTIK* 2017 626–631.

[B122] Wang C. Zou X. Tang Y. Luo L. Feng W. (2016). Localisation of litchi in an unstructured environment using binocular stereo vision. *Biosyst. Eng.* 145 39–51.

[B123] Wang Y. Yang Y. Yang C. Zhao H. Chen G. Zhang Z. (2019). End-effector with a bite mode for harvesting citrus fruit in random stalk orientation environment. *Comput. Electr. Agricult.* 157 454–470.

[B124] Wang Z. (2018). “Robot obstacle avoidance and navigation control algorithm research based on multi-sensor information fusion,” in *Proceedings of the 11th International Conference on Intelligent Computation Technology and Automation (ICICTA)*, Changsha, 351–354.

[B125] Wang Z. Walsh K. Verma B. (2017). On-tree mango fruit size estimation using RGB-D images. *Sensors* 17 2738. 10.3390/s17122738 29182534 PMC5751737

[B126] Watts P. L. Lewis A. Nagpal B. K. (1983). “Economic considerations in industrial robotics,” in *Proceedings of the Twenty-third International Machine Tool Design and Research Conference*, ed. Davies B. J. (London: Palgrave), 527–532.

[B127] Wei X. Jia K. Lan J. Li Y. Zeng Y. Wang C. (2014). Automatic method of fruit object extraction under complex agricultural background for vision system of fruit picking robot. *Optik* 125 5684–5689.

[B128] Wibowo T. S. Sulistijono I. A. Risnumawan A. (2016). “End-to-end coconut harvesting robot,” in *Proceedings of the in 18th IEEE International Electronics Symposium (IES)*, Warwick, 444–449.

[B129] Williams H. Ting C. Nejati M. Jones M. H. Penhall N. Lim J. (2019). Improvements to and large-scale evaluation of a robotic kiwifruit harvester. *J. Field Robot.* 37 1–15.

[B130] Williams H. A. M. Jones M. H. Nejati M. Seabright M. J. Bell J. Penhall N. D. (2019). Robotic kiwifruit harvesting using machine vision, convolutional neural networks, and robotic arms. *Biosyst. Eng.* 181 140–156.

[B131] Xiang R. Jiang H. Ying Y. (2014). Recognition of clustered tomatoes based on binocular stereo vision. *Comput. Electr. Agricult.* 106 75–90.

[B132] Xiong J. He Z. Lin R. Liu Z. Bu R. Yang Z. (2018a). Visual positioning technology of picking robots for dynamic litchi clusters with disturbance. *Comput. Electr. Agricult.* 151 226–237.

[B133] Xiong J. Lin R. Liu Z. He Z. Tang L. Yang Z. (2018b). The recognition of litchi clusters and the calculation of picking point in a nocturnal natural environment. *Biosyst. Eng.* 166 44–57.

[B134] Xiong Y. Peng C. Grimstad L. From P. J. Isler V. (2019). Development and field evaluation of a strawberry harvesting robot with a cable-driven gripper. *Comput. Electr. Agricult.* 157 392–402.

[B135] Xu W. Chen H. Su Q. Ji C. Xu W. Memon M. (2019). Shadow detection and removal in apple image segmentation under natural light conditions using an ultrametric contour map. *Biosyst. Eng.* 184 142–154.

[B136] Xue J. Zhang L. Grift T. E. (2012). Variable field-of-view machine vision based row guidance of an agricultural robot. *Comput. Electr. Agricult.* 84 85–91.

[B137] Yamamoto K. Guo W. Yoshioka Y. Ninomiya S. (2014). On plant detection of intact tomato fruits using image analysis, and machine learning methods. *Sensors* 14 12191–12206. 10.3390/s140712191 25010694 PMC4168514

[B138] Yang D. Li H. Zhang L. (2016). Study on the fruit recognition system based on machine vision. *Adv. J. Food Sci. Technol.* 10 18–21.

[B139] Yang G. Bellingham J. Dupont P. E. Fischer P. Floridi L. Full R. (2018). The grand challenges of science robotics. *Sci. Robot.* 3 1–14. 10.1126/scirobotics.aar7650 33141701

[B140] Yin H. Chai Y. Yang S. X. Mittal G. S. (2009). “Ripe tomato extraction for a harvesting robotic system,” in *Proceedings of the IEEE International Conference on Systems Man and Cybernetics Conference Proceedings*, San Antonio, TX, 2984.

[B141] Yin J. Mao H. Zhong S. (2009). Segmentation methods of fruit image based on color difference. *J. Commun. Comput.* 6 40–45.

[B142] Yu Y. Sun Z. Zhao X. Bian J. Hui X. (2018). “Design and implementation of an automatic peach-harvesting robot system,” in *Proceedings of the 2018 Tenth International Conference on Advanced Computational Intelligence (ICACI)*, Xiamen, 700–705.

[B143] Zhang P. Xu L. (2018). Unsupervised segmentation of greenhouse plant images based on statistical method. *Sci. Rep.* 8:4465. 10.1038/s41598-018-22568-3 29535402 PMC5849718

[B144] Zhang Q. Karkee M. Tabb A. (2019). “The use of agricultural robots in orchard management,” in *Robotics and Automation for Improving Agriculture*, Ed. Billingsley J. (Cambridge: Burleigh Dodds Science Publishing), 187–214.

[B145] Zhang Y. Li M. Qiao J. Liu G. (2008). A segmentation algorithm for apple fruit recognition using artificial neural network. *Acta Optica Sin.* 28 2104–2108.

[B146] Zhao D. Lv J. Ji W. Zhang Y. (2011). Design and control of an apple harvesting robot. *Biosyst. Eng.* 110 112–122.

[B147] Zhao J. Tow J. Katupitiya J. (2005). “On-tree fruit recognition using texture properties and color data,” in *Proc. 2005 IEEE/RSJ International Conference on Intelligent Robots and Systems*, Piscataway, NJ, 263–268.

[B148] Zhao Y. Gong L. Huang Y. Liu C. (2016). A review of key techniques of vision-based control for harvesting robot. *Comput. Electr. Agricult.* 127 311–323.

[B149] Zhuang J. Hou C. Tang Y. He Y. Guo Q. Zhong Z. (2019). Computer vision-based localisation of picking points for automatic litchi harvesting applications towards natural scenarios. *Biosyst. Eng.* 187 1–20.

[B150] Zhuang J. J. Luo S. M. Hou C. J. Tang Y. He Y. Xue X. Y. (2018). Detection of orchard citrus fruits using a monocular machine vision-based method for automatic fruit picking applications. *Comput. Electr. Agricult.* 152 64–73.

[B151] Zou X. Ye M. Luo C. Xiong J. Luo L. Wang H. (2016). Fault-tolerant design of a limited universal fruit-picking end-effector based on vision-positioning error. *Appl. Eng. Agricult.* 32 5–18.

[B152] Zou X. Zou H. Lu J. (2012). Virtual manipulator-based binocular stereo vision positioning system and errors modelling. *Mach. Vis. Appl.* 23 43–63.

